# Relationship between comorbidities and treatment decision-making in elderly hip fracture patients

**DOI:** 10.1007/s40520-019-01134-5

**Published:** 2019-04-16

**Authors:** Jinxing Wei, Li Zeng, Shitong Li, Fang Luo, Zhou Xiang, Qunfang Ding

**Affiliations:** 1 Department of Geriatrics, West China Hospital, Sichuan University, NO. 37 GuoXue Road, Chengdu, 610041 Sichuan China; 2 Department of Geriatrics, Sichuan Provincial People’s Hospital, Chengdu, 610072 China

**Keywords:** Aging, Hip fracture, Comorbidity, Charlson Comorbidity Index (CCI), Treatment decision-making

## Abstract

**Background:**

Elderly patients are at a higher risk for hip fracture. Moreover, hospitalized elderly patients with hip fracture are vulnerable to adverse outcomes including higher mortality rate and long-term disability. Treatment decision-making with respect to surgical procedure and perioperative management of these patients is typically challenging owing to the presence of multiple comorbid conditions.

**Aims:**

The purpose of this study was to investigate the relationship between comorbidities in elderly patients with hip fracture and the treatment decision-making.

**Methods:**

884 geriatric patients (age ≥ 60 years) with hip fracture were included. Comorbidities related to age were measured using the Charlson Co-morbidity Index (CCI) and age-adjusted CCI. The CCI of each geriatric hip fracture patient was calculated based on data retrieved from the medical records. The relationship of CCI and age-adjusted CCI with surgical procedure, time-to-surgery, length of hospital stay, and perioperative management (transfusion, anti-coagulation, and analgesia) was assessed.

**Results:**

Mean age of patients was 78.01 ± 8.62 years. The mean CCI was 0.79 ± 0.036; the mean age-adjusted CCI was 4.15 ± 0.047. The CCI was significantly associated with time-to-surgery (*P* = 0.004), surgical treatment (*P* < 0.001), and transfusion (*P* = 0.023). The age-adjusted CCI was significantly associated with surgical treatment (*P* < 0.001), analgesia (*P* = 0.003) and transfusion (*P* < 0.001). The length of hospital stay was associated with both CCI (*P* = 0.041), age-adjusted CCI (*P* = 0.002), and hypertension (*P* = 0.012). Hospital expenses showed a significant association with CCI (*P* = 0.000), age-adjusted CCI (*P* = 0.029), osteoprosis (*P* = 0.007), and hypertension (*P* = 0.001).

**Conclusion:**

In this study, comorbidities were positively associated with surgical procedure and perioperative management of elderly patients with hip fracture.

**Electronic supplementary material:**

The online version of this article (10.1007/s40520-019-01134-5) contains supplementary material, which is available to authorized users.

## Introduction

Hip fractures are a major cause of mortality and disability in the elderly population. Moreover, it increases the burden on caregivers, families, as well as the society at large. The number of geriatric patients with hip fracture has dramatically increased over the last decade [1–3]. Hip fractures are the second most common cause of hospitalization of elderly patients, with an estimated annual cost of over $650 million [3, 4]. Elderly patients with hip fractures constitute the most vulnerable group among hospitalized patients, and their clinical outcomes are closely linked to the quality of care [5].

In the last decade, several studies have sought to identify factors that influence the clinical outcomes of these patients [6, 7]. Timing of surgery as well as other medical factors have been shown to influence the clinical outcomes of geriatric patients with hip fracture [8]. Surgical delay may lead to an increase in mortality and adverse clinical outcomes such as infection and pressure sores [9, 10]. Therefore, it is important to evaluate the patient’s condition for making an individual management plan. Clinical guidelines recommend that reparative surgery should be performed within 24–48 h of hospital admission;[7, 8] however, adequate perioperative assessment of the patient condition including evaluation of comorbid diseases is necessary to develop an optimal treatment plan for elderly patients.

When compared with elderly hip fracture patients without comorbidities, those with comorbidities may accept less-aggressive medical therapy, and experience extended duration of hospitalization, decreased quality of life, and increased health care costs. This is because prolonged immobilization is likely to increase the incidence of urinary and pulmonary complications [11]. Thus, the combination of advanced age and presence of comorbidities in elderly patients with hip fracture presents a considerable challenge with respect to surgical decision-making and perioperative management [12, 13].

The primary objective of this study was to investigate the association between comorbidities [as assessed by Charlson Comorbidity Index (CCI) and age-adjusted CCI] [14, 15] and treatment decision-making including the surgical procedure and perioperative management in elderly patients with hip fracture.

## Methods

### Study design and population

This was a cross-sectional observational study of patients with femoral neck fracture or intertrochanteric fracture who were admitted to the West China Hospital of Sichuan University, Chengdu, China between January 2011 and December 2015. The inclusion criteria were: patients aged ≥ 60 years with femoral neck fracture or intertrochanteric fracture diagnosed by computed tomography (CT) or X-ray for the first time; presence of comorbid conditions (Table 2); time interval from fracture to hospitalization ≤ 15 days. The exclusion criteria were: patients with pathological fracture; incomplete medical records; patients with a prior history of hip fracture, and previous surgical management.

The study was approved by the Biomedical Ethics Committee of the West China Hospital of Sichuan University. Written informed consent was obtained from all subjects.

### Data collection

This study was a purely descriptive study of consecutive patients who were registered in the information system of the West China hospital. Data pertaining to the following variables were collected from the medical records: established diagnosis, age at admission, gender, cause of fracture, type of fracture, time-to-surgery, length of hospital stay, hospitalization expenses, comorbid conditions, treatment details, and postoperative complications.

Data collection was performed by postgraduate medical students at the West China Clinical Medical School, West China Hospital, Sichuan University. All data collectors underwent training for data extraction and data entry.

### Assessment of comorbidities

The Charlson Comorbidity Index (CCI) was used to evaluate patient comorbidity. The CCI incorporates the number of pre-existing comorbid conditions and their severity [14, 15]. It represents the sum of weighted index for different subset of conditions: one point was awarded for myocardial infarction, congestive heart failure, peripheral vascular disease, cerebrovascular disease (except hemiplegia), dementia, chronic pulmonary disease, connective tissue disorders, ulcer disease, mild liver disease, and diabetes without complications. Two points were awarded for diabetes with end-organ damage, hemiplegia, moderate or severe renal disease, or any malignancy including leukemia, lymphoma, diabetes with chronic complications, and solid tumors (non metastatic). Three points were awarded for moderate or severe liver disease, while six points were awarded for acquired immune deficiency syndrome (AIDS) and metastatic solid tumors. Higher scores correspond to increased risk of mortality. The total score was calculated for each patient; based on the total score, the study population was classified into four ordinal categories: 0, 1–2, 3–4, ≥ 5 points. In addition, age-adjusted CCI was also calculated, wherein one point was awarded for each decade of life after the age of 50 years [16].

Hypertension and osteoporosis, which are not included in the CCI, were also included in the analysis. Hypertension was defined as a systolic blood pressure > 140 mmHg and/or diastolic blood pressure > 90 mmHg or by history of antihypertensive therapy. Patients who fulfilled one out of the following three criteria were considered osteoporotic: (1) occurrence of adulthood hip or vertebral fracture in the absence of major trauma; (2) *T* score < − 2.5 on dual energy X-ray absorptiometry (DXA); (3) presence of fragility fractures of proximal humerus, pelvis, or distal forearm with − 2.5 < *T* score < − 1.0.

Data pertaining to chronic diseases that were already diagnosed were obtained from the medical records. The differential diagnosis had been completed in the past.

### Statistical analysis

Continuous variables are expressed as mean ± standard deviation (SD) and compared using Student’s *t* test. Categorical variables are expressed as percentages and compared using the Chi-squared test. The CCI and other comorbidities were used as independent variables. Binary logistic regression models were used to examine the relationship of CCI with complications, surgical choice, time-to-surgery, transfusion, anti-coagulation, analgesia, and complications after hip fractures. Linear regression analysis was performed to examine the relationship of length of hospital stay and hospital expenses with CCI, age-adjusted CCI, osteoporosis, and hypertension. The results are presented as crude and adjusted regression coefficients and 95% confidence intervals, standardized coefficients, and *P* values. All statistical tests of significance were two-tailed, and *P* values < 0.05 were considered statistically significant. Statistical analyses were performed using SPSS 16.0 statistics software (SPSS Inc. Chicago, IL, USA).

## Results

A total of 884 patients with hip fracture were included in the study (Table 1). Of these 558 (66.5%) were female and 296 (33.5%) were male; the mean age of patients was 78.01 ± 8.62 years. A vast majority of the patients had fragility fractures [839 (95.0%) patients]. Patients with femoral neck fracture accounted for 60.2% of the study population. The average CCI and age-adjusted CCI were 4.15 ± 0.047 and 0.79 ± 0.036, respectively; most of the patients had a CCI in the range of 0–2 points. Male patients exhibited a tendency for higher CCI than female patients (*P* = 0.053). The age-adjusted CCI for male patients was significantly higher than that for female patients (*P* = 0.019). The mean age-adjusted CCI of patients with intertrochanteric fracture was significantly higher than that of patients with femoral neck fracture (*P* = 0.010) (Table 1).

**Table 1:** Summary statistics of patient characteristics

Variables	Total (*n* = 884)	CCI by categories				Age-adjusted CCI	*P*	CCI	*P*
		0	1–2	3–4	≥ 5				
Gender, *n* (%) or mean ± SD									
Male	296 (33.5)	136 (30.0)	130 (35.8)	26 (44.1)	3 (42.9)	4.31 ± 1.46	0.019	0.88 ± 0.64	0.053
Female	588 (66.5)	318 (70.0)	233 (64.2)	33 (55.9)	4 (57.1)	4.07 ± 1.48		0.74 ± 0.43	
Age, *n* (%) or mean ± SD									
60–69	157 (17.7)	90 (19.8)	55 (15.2)	9 (15.3)	2 (28.6)	2.71 ± 1.12	< 0.001	0.71 ± 1.12	0.620
70–79	325 (36.8)	163 (35.9)	138 (38.0)	23 (39.0)	1 (14.3)	3.79 ± 1.04		0.79 ± 1.04	
80–89	329 (37.2)	162 (35.7)	147 (40.5)	18 (30.5)	2 (28.6)	4.80 ± 0.57		0.80.±1.03	
≥ 90	73 (8.3)	39 (8.6)	23 (6.3)	9 (15.3)	2 (28.6)	5.96 ± 1.55		0.90 ± 1.29	
Fracture reasons, *n* (%) or mean ± SD									
Trauma fracture	44 (5.0)	22 (4.8)	20 (5.5)	2 (3.4)	0 (0)	3.37 ± 1.23	0.436	0.64 ± 0.78	0.089
Fragility fracture	839 (95.0)	432 (95.2)	343 (94.5)	57 (96.6)	7 (100.0)	4.18 ± 0.049		0.80 ± 0.037	
Fracture patterns, *n* (%) or mean ± SD									
Femoral neck fracture	532 (60.2)	171 (37.7)	143 (39.4)	32 (54.2)	5 (71.4)	3.94 ± 1.32	< 0.001	0.70 ± 0.98	0.002
Intertrochanteric fracture	352 (39.8)	283 (62.3)	220 (60.6)	27 (45.8)	2 (28.6)	4.47 ± 1.50		0.92 ± 1.18	

The prevalence of comorbidities and complications in our study population is presented in Table 2. Most of the patients scored one point for chronic diseases; there was a high proportion of patients with chronic pulmonary disease (11.65%) and diabetes without complications (22%). The prevalence of hypertension and osteoporosis was 45.02% and 43.67%, respectively. The most common complication after hip fracture was pulmonary infection (18.2%). The results of univariate logistic regression analysis between age-adjusted CCI, CCI, hypertension, and osteoporosis are presented in Table 3. Both age-adjusted CCI and CCI showed a significant association with higher incidence of pulmonary infection (OR 1.448, *P* = 0.000, 95% CI 1.287–1.629) after hip fracture. The CCI had a tendency to associate with venous thrombosis and urinary tract infection. However, age-adjusted CCI was a predictor of venous thrombosis (OR 1.192, *P* = 0.024, 95% CI 1.023–1.387) and urinary tract infection (OR 1.286, *P* = 0.043, 95% CI 1.008–1.639). Additionally, hypertension contributed significantly to the increase in venous thrombosis (OR 1.652, *P* = 0.036, 95% CI 1.034–2.652). Conversely, osteoporosis was not associated with complications after hip fracture (Table 3).

**Table 2:** Description of the comorbidities and post-hip fracture complications in the study population

Variables	Points	*N* (%)
Comorbidities taken into account in the CCI		
Chronic pulmonary disease	1	103 (11.65)
Connective tissue diseases	1	20 (2.3)
Congestive heart failure	1	32 (3.6)
Cerebrovascular disease (without hemiplegia)	1	57 (6.4)
Dementia	1	35 (3.9)
Diabetes without complications	1	195 (22)
Mild liver disease	1	9 (1.0)
Myocardial infarction	1	4 (0.4)
Peripheral vascular insufficiency	1	2 (0.2)
Ulcer disease	1	3 (0.3)
Diabetes with chronic complications	2	5 (0.5)
Moderate-to-severe renal disease	2	34 (3.8)
Hemiplegia	2	14 (1.6)
Tumor including leukemia and lymphoma	2	38 (4.3)
Hepatopathies at severe stage	3	2 (0.2)
AIDS	6	1 (0.1)
Metastatic cancer	6	1 (0.1)
Presence of other comorbidities		
Hypertension	–	398 (45.02)
Osteoporosis	–	386 (43.67)
Complications after hip fracture	–	
Venous thrombosis	–	78 (8.8)
Urinary tract infection	–	25 (2.8)
Pulmonary infection	–	161 (18.2)
Delirium	–	14 (1.6)
Pressure sores	–	29 (3.2)

*CCI* Charlson Comorbidity Index, *AIDS* acquired immune deficiency syndrome

**Table 3:** Results of logistic regression analysis showing the association of complications after hip fracture with age-adjusted CCI, CCI, hypertension, and osteoporosis

	Age-adjusted CCI				CCI			
	*B*	*P*	Odds ratio	95% confidence interval	*B*	*P*	Odds ratio	95% confidence interval
Venous thrombosis	0.175	0.024	1.192	1.023–1.387	0.176	0.069	1.193	0.986–1.443
Urinary tract infection	0.251	0.043	1.286	1.008–1.639	0.237	0.119	1.268	0.941–1.708
Pulmonary infection	0.370	0.000	1.448	1.287–1.629	0.262	0.000	1.300	1.126–1.500
Delirium	0.160	0.355	1.173	0.836–1.646	− 0.002	0.993	0.998	0.609–1.636
Pressure sore	− 0.045	0.741	0.956	0.730–1.251	− 0.176	0.392	0.838	0.560–1.256

	Hypertension				Osteoporosis			
	*B*	*P*	Odds ratio	95% confidence interval	*B*	*P*	Odds ratio	95% confidence interval
Venous thrombosis	0.502	0.036	1.652	1.034–2.652	0.393	0.099	1.481	0.929–2.359
Urinary tract infection	− 0.043	0.917	0.958	0.430–2.135	− 0.155	0.708	1.268	0.380–1.928
Pulmonary infection	− 0.138	0.432	0.871	0.617–1.230	0.144	0.409	1.155	0.820–1.628
Delirium	− 0.394	0.480	0.483	0.224–2.028	0.259	0.631	1.296	0.451–3.725
Pressure sore	− 0.457	0.250	0.633	0.291–1.378	− 0.097	0.801	0.908	0.428–1.924

*CCI* Charlson Comorbidity Index

In logistic regression, the CCI remained remarkably associated with time-to-surgery (OR 0.715, *P* = 0.004, 95% CI 0.567–0.901), surgical choice (OR 0.759, *P* = 0.000, 95% CI 0.666–0.867), and transfusion (OR 1.175, *P* = 0.023, 95% CI 1.022–1.350) (Table 4). The age-adjusted CCI showed a significant positive association with surgical choice (OR 0.704, *P* = 0.000, 95% CI 0.633–0.783) and transfusion (OR 1.252, *P* = 0.000, 95% CI 1.122–1.396).

**Table 4:** Logistic regression of the age-adjusted CCI, CCI, and treatments

		Age-adjusted CCI				CCI			
		*B*	*P*	Odds ration	95% confidence interval	*B*	*P*	Odds ratio	95% confidence interval
Time-to-surgery									
≤ 48 h	145 (23.1)	− 0.36	0.621	0.965	0.837–1.112	− 0.336	0.004	0.715	0.567–0.901
> 48 h	483 (76.9)								
Whether to operate									
Yes	628 (71.0)	− 0.351	0.000	0.704	0.633–0.783	− 0.275	0.000	0.759	0.666–0.867
No	256 (29.0)								
Surgical treatment									
Osteosynthesis	355 (56.5)	− 0.084	0.171	0.919	0.815–1.037	− 0.139	0.104	0.870	0.736–1.029
Arthroplasty	273 (43.5)								
Analgesia									
Yes	594 (67.2)	− 0.153	0.002	0.859	0.778–0.947	− 0.037	0.573	0.963	0.779–0.949
No	290 (32.8)								
Transfusion									
Yes	194 (21.9)	0.225	0.000	1.252	1.122–1.396	0.161	0.023	1.175	1.022–1.350
No	690 (78.1)								
Anti-coagulation									
Yes	544 (66.9)	− 0.044	0.361	0.959	0.896–1.025	− 0.034	0.596	0.967	0.852–1.096
No	342 (33.1)								

*CCI* Charlson Comorbidity Index

On linear regression analysis, the length of hospital stay was associated with CCI (*B* 1.659, *P* = 0.041, 95% CI 0.814–2.503), age-adjusted CCI (*B* 0.938, *P* = 0.002, 95% CI 0.297–1.580), and hypertension (*B* 2.339, *P* = 0.012, 95% CI 0.513–4.164). Hospital expenses showed a significant association with CCI (*B* 2963.144, *P* = 0.000, 95% CI 1336.728–4589.561), age-adjusted CCI (*B* 1384.695, *P* = 0.029, 95% CI 145.561–2623.829), osteoporosis (*B* 4811.431, *P* = 0.007, 95% CI 1290.779–8332.084), and hypertension (*B* 5822.974, *P* = 0.001, 95% CI 2319.887–9326.061) (Table 5).

**Table 5:** Correlation of Charlson Comorbidity scores, hypertension, osteoporosis with length of hospital stay and hospital expenses

	Age-adjusted CCI				CCI			
	*B*	*P*	Beta	95% CI	*B*	*P*	Beta	95% CI
Length of hospital stay	0.938	0.004	0.096	0.297–1.580	1.659	0.000	0.129	0.814–2.503
Hospital expenses	1384.695	0.029	0.074	145.561–2623.829	2963.144	0.000	0.120	1336.728–4589.561

	Osteoprosis				Hypertension			
	*B*	*P*	Beta	95% CI	*B*	*P*	Beta	95% CI
Length of hospital stay	14.229	0.073	0.060	13.017–15.441	2.339	0.012	0.930	0.513–4.164
Hospital expense	4811.431	0.007	0.090	1290.779–8332.084	5822.974	0.001	0.109	2319.887–9326.061

*CCI* Charlson Comorbidity Index, *CI* confidence interval

## Discussion

In the present study, 76.9% of geriatric patients with hip fracture were not operated during the first 48 h. This is likely attributable to the time required for assessment and stabilization of comorbid diseases. The weight of comorbidities in elderly patients with hip fracture (as assessed by CCI) was associated with time-to-surgery in excess of 48 h, which implies that increase in the CCI increased the risk of delayed surgery. However, it showed no significant association with time-to-surgery in excess of 48 h after adjusting for age. Additionally, the weight of comorbidities in elderly patients with hip fracture (as assessed by age-adjusted CCI) was associated with unavailable operation, increased transfusion, increased hospital stay, and hospital expenses.

To the best of our knowledge, this is the first study that investigated the possible association of comorbidities in elderly patients with hip fracture and their treatment preferences, especially with respect to surgical timing and approach. Several studies have shown that patients with hip fracture who are operated within 48 h experience better clinical outcomes and functional recovery [17–20]. Moreover, clinical guidelines also recommend that surgery for geriatric patients with hip fracture should be performed within the first 48 h of hospital admission [21, 22]. A study of Spanish patients older than 65 years with hip fracture, conducted by Sanz-Reig et al. [23] investigated preoperative risk factors for surgical delay of > 2 days from admission. They found that only 44.1% of patients were operated within the first 2 days of admission. Moreover, CCI > 2 (mean CCI: 2.8 ± 1.8) was independently associated with surgical delay of > 2 days.

However, in a study of 56,500 patients with hip fracture (public hospitals across 8 Spanish regions during 2002–2005) conducted by Librero et al. [24] early surgery was conducted on 25% of patients older than 60 years; the time-to-surgery assessed by the Risk Mortality Index was associated with higher mortality, but not with higher chronic comorbidities, male gender, or older age. Hence, comorbidity status was a good preoperative indicator of short-term and long-term mortality risk in geriatric patients with hip fracture [13]. Compared with these two studies [23, 24], only 23.1% of patients were operated within the first 48 h of admission, which is a very low percentage. Additionally, the lower average CCI in our study population was likely attributable to the lower average age of patients. In the study by Librero et al., two-thirds of the patients were aged ≥ 80 years; in another Spanish study, the average age of patients was 83.7 ± 6.9 years. The second potential reason may be the higher proportion of female patients in our study, who had lower average age, average CCI, and average age-adjusted CCI. About 47.6% female patients in our study had osteoporosis, while the corresponding percentage among male patients was 35.8% only (Supplementary Table 1); it seems that osteoporosis was a major cause of hip fracture among female patients, while chronic diseases and aging played a more important role in male patients. Therefore, the higher proportion of female patients may have induced lower average CCI and average age-adjusted CCI. The last reason was the high proportion of patients with CCI in the range of 0–2.

Several strengths of the present study should be noted. Identification of the weight of patients’ comorbidities may have been an important contributor to the delay in surgery when the surgeons were unavailable. A study conducted by Gretchen M. Oroszfor quantified the time interval between injury and hospitalization in older patients with hip fracture [25]; they found that it was common to delay surgery by more than 24 h from hospitalization [25]. In some cases, the delay was related to patients themselves while in other cases the delay was attributed to systemic factors. Another strength is the assessment of the association of patients’ comorbidities with the surgical procedure and perioperative management in geriatric patients with hip fracture, rather than collecting these information only from medical history. Lastly, the number of geriatric patients with hip fracture was large enough for conducting accurate evaluation of the relationship between comorbidities and treatment decision-making.

Surgical intervention in geriatric patients with hip fracture has been shown to decrease the risk of mortality up to 1 year, prevent progression to disability, and help maintain functional status of the patient [26–28]. Kau et al. [29] conducted a prospective cohort study of 212 geriatric patients (age > 60 years) with hip fractures in Singapore. They observed that conservatively managed patients had higher CCI scores for mortality than surgically treated patients, which is consistent with our findings. Roubinian et al. [30] retrospectively evaluated the role of disease severity and comorbidity for predicting inpatient red blood cell transfusion events in 275,874 patients (444,969 hospitalizations); they found that disease severity and comorbidities play a limited role in predicting the likelihood of transfusion at the time of admission. Based on these observations, the incidence of transfusion relative to patient comorbidities in our study may be explained by inclusion of patients > 60 years of age.

Several limitations of our study should be taken into account while interpreting the findings. First, the present study was a cross-sectional observational study; hence, the influence of confounding factors on our results cannot be ruled out. Secondly, there is lack of a unified comorbidity index for assessment of comorbidities in geriatric patients with hip fracture,[31] which may affect the reliability of our findings. We used the CCI and age-adjusted CCI, which is currently considered as the gold standard for assessment of comorbidity. In addition, complete data pertaining to treatment of comorbid conditions were not available; several studies have shown that the effectiveness of treatment of comorbidities affects the outcomes of elderly patients with hip fractures and the mean value of CCI was lower with respect to the majority of these studies [32–34]. Therefore, further multicenter randomized controlled studies with complete data and a validated comorbidity index are required to solve the aforementioned issues.

## Conclusion

In this study, comorbidities were positively associated with the surgical procedure and perioperative management in elderly patients with hip fracture.

## Electronic supplementary material

Below is the link to the electronic supplementary material.

Supplementary material 1 (DOCX 23 KB)

## Data Availability

The datasets generated during and/or analysed during the current study are available from the corresponding author on reasonable request.
