# Supplementary material for: Relationship between comorbidities and treatment decision-making in elderly hip fracture patients
Source: Aging Clin Exp Res. 2019 Apr 16;31(12):1735–41. doi: 10.1007/s40520-019-01134-5 (PMC6825646; doi:10.1007/s40520-019-01134-5)
Supplement: Supplementary file 1 — Supplementary material 1 (DOCX 23 KB) [file 40520_2019_1134_MOESM1_ESM.docx]

Supplementary Table 1.

Variables Women Men *P*

Age, mean±SD 77.71±8.463 78.58±8.889 0.157

Age-adjusted CCI, mean±SD 4.07±1.46 4.31±1.48 0.019

CCI, mean±SD 0.74±0.43 0.88±0.64 0.053

Presence of other comorbidities, N (%)

Hypertension 270 (45.09) 128 (43.2%) 0.474

Osteoporosis 280 (47.6) 106 (35.8%) 0.001

CCI, Charlson comorbidity index; AIDS, acquired immune deficiency syndrome; SD, standard deviation

Supplementary Table 2. Results of logistic regression analysis showing association of osteoporosis with age-adjusted CCI and CCI

Age-adjusted CCI CCI

*B P Odds 95% Confidence B P Odds 95% Confidence*

*Ratio Interval Ratio Interval*

Osteoporosis 0.108 0.025 1.1141.014–1.224-0.0480.4510.953 0.841–1.080

CCI, Charlson comorbidity index
